# Lithium-induced Hyperparathyroidism and Hypercalcemia

**DOI:** 10.7759/cureus.4590

**Published:** 2019-05-02

**Authors:** Srikanth Naramala, Hussain Dalal, Sreedhar Adapa, Amir Hassan, Venu Madhav Konala

**Affiliations:** 1 Rheumatology, Adventist Medical Center, Hanford, USA; 2 Internal Medicine, Internal Medicine Multi-Specialty Clinic, Houston, USA; 3 Nephrology, The Nephrology Group, Visalia, USA; 4 Endocrinology, Memorial Hermann Hospital, Houston, USA; 5 Internal Medicine, Ashland Bellefonte Cancer Center, Ashland, USA

**Keywords:** lithium, hyperparathyroidism, hypercalcemia

## Abstract

Lithium is one of the mainstays of treatment for bipolar disorder. Chronic lithium therapy can rarely lead to hypercalcemia secondary to lithium-induced hyperparathyroidism. We present a 66-year-old female patient with bipolar disorder on lithium therapy presenting with hypercalcemia. We discussed the pathophysiology and management of hyperparathyroidism and hypercalcemia in patients on chronic lithium therapy.

## Introduction

Lithium has been one of the mainstays of treatment in patients who are diagnosed with bipolar disorder. Although the drug has a varied side-effect profile, one of the rare side effects, which is often overlooked, is its ability to cause hyperparathyroidism. Long-term hyperparathyroidism and subsequent hypercalcemia are known to cause the classic “stones (renal calculi), bones (osteoporosis), abdominal groans (pancreatitis), thrones (constipation) and psychiatric overtones (delirium).” While the cessation of lithium is the definitive therapy for reversing these side effects, parathyroidectomy, along with the cessation of lithium use, is the only cure for hyperparathyroidism.

## Case presentation

**Figure 1:**
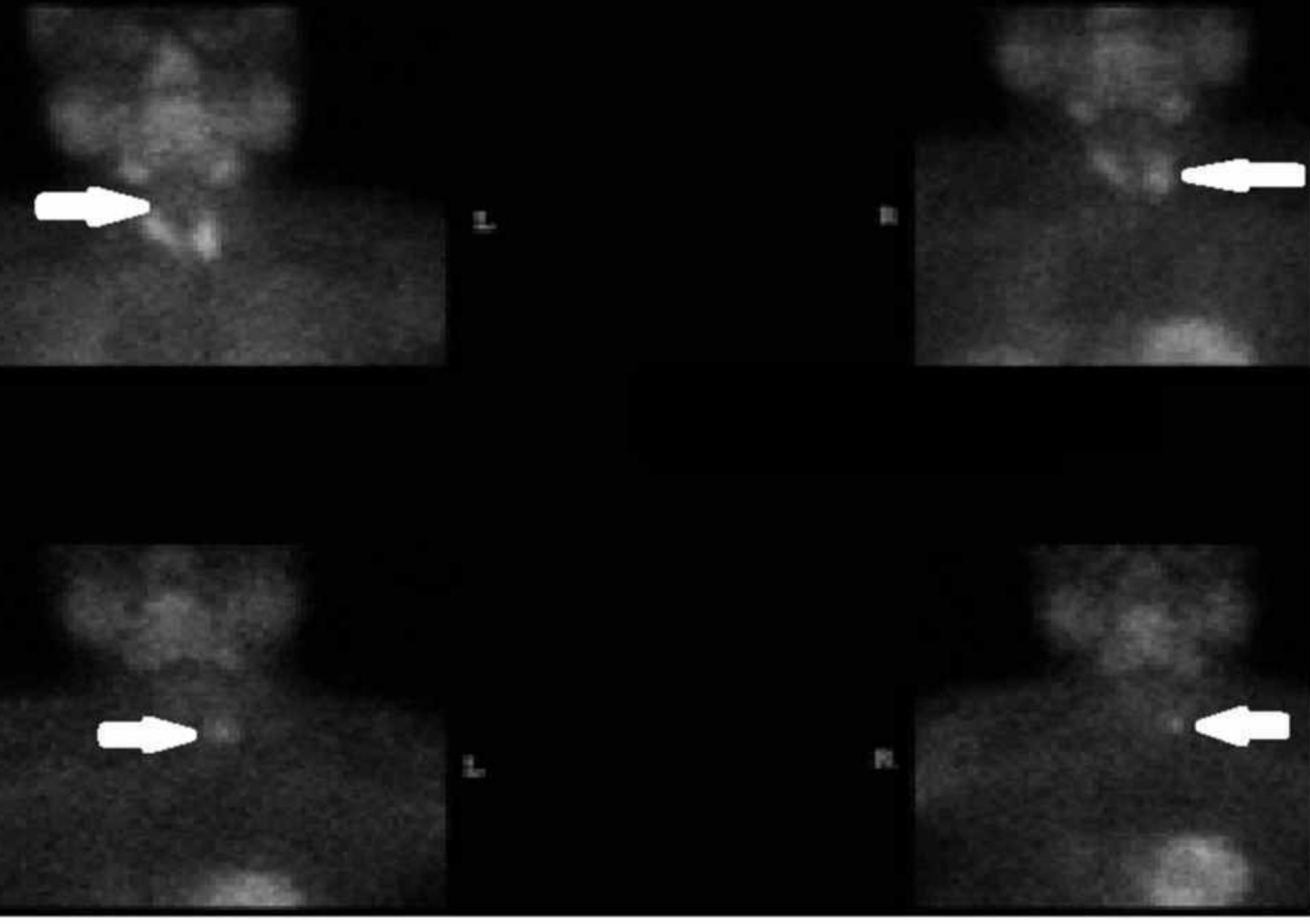
Parathyroid scan showing uptake in all sites at 10 minutes (top two images) and a left-sided adenoma at four hours (bottom two images) - washout over time of sestamibi from the normal PTH tissue more rapid than a PTH adenoma. Persistent uptake in the left PTH area suggesting an adenoma on the four-hour delayed scan. PTH: parathyroid hormone

A 66-year-old Caucasian female with a history of bipolar disorder, maintained on lithium therapy for two years, presented to our practice for hypercalcemia. Review of systems was negative for bone pain, abdominal pain, and any psychiatric findings. Her total serum calcium (after correction) was found to be 11.58 mg/dl, ionized calcium was found to be 6.2 mg/dl, and parathyroid hormone (PTH) was found to be elevated at 100 pg/ml. Bone density was normal. The parathyroid scan showed a functioning, 2 cm, right parathyroid adenoma and she underwent a right parathyroidectomy. Postoperatively, her PTH levels returned to normal. Six months postoperatively, routine blood work found elevated serum calcium levels. A repeat parathyroid scan showed increased uptake in the left parathyroid lobe consistent with a parathyroid adenoma (Figure 1).

The patient underwent a second parathyroidectomy of the left parathyroid gland. Her postoperative PTH levels were within normal limits. It was concluded that lithium was the contributing factor to parathyroid adenomas casing hyperparathyroidism. Lithium therapy was discontinued under the guidance of a psychiatrist. At the six-month follow-up, her PTH and calcium levels were found to be within normal limits.

## Discussion

Lithium, which has been in clinical use since 1950 [1-2], is one of the cornerstone treatments for bipolar disorder. Although lithium salts are known to disrupt thyroid function [3], lithium-induced parathyroid dysfunction is rare. The first case of lithium-induced hyperparathyroidism was reported in 1973 [1]. Although primary hyperparathyroidism usually arises from parathyroid adenomas, it is also essential to consider other factors that could be contributing to primary hyperparathyroidism.

A proposed mechanism of how lithium causes hyperparathyroidism points to the finding that lithium can alter the set point of receptors that sense calcium in parathyroid cells, thus promoting excess parathyroid release [1]. The extracellular calcium-sensing receptor (CaSR) has a vital role in serum calcium homeostasis by regulating PTH levels in the blood. CaSR plays a significant role in PTH synthesis, PTH secretion, and cellular proliferation. Calcium/inorganic phosphate equilibrium, cation transport, production of renin, and urine concentration are some of the pivotal actions of widespread CaSR along the nephron.

In addition to altering the set points of CaSR, findings also suggest that lithium antagonizes the CaSR, which raises the threshold level of calcium required to suppress PTH output by the parathyroid [4]. Consistently elevated PTH levels lead to hypercalcemia. As is well-known, the long-term effects of hypercalcemia are manifested in the form of renal calculi, osteoporosis, and the gastrointestinal side effects of constipation, anorexia, nausea, and vomiting [5]. Cardiac manifestations include the shortening of the QT interval and deposition of calcium in the heart valves [5]. Calcium levels should be periodically monitored in patients on long-term lithium therapy. Parathyroidectomy is the mainstay of treatment in a functioning parathyroid adenoma, and in cases where an external factor like lithium is involved, stopping the offending agent is the definitive cure.

## Conclusions

One of the notable side effects of lithium use is its ability to cause hyperparathyroidism. Chronic hyperparathyroidism with hypercalcemia causes a myriad of pathologies involving multiple organ systems. Surgical excision of lithium-induced parathyroid adenomas and the cessation of lithium should be the definitive management in these patients. After stopping lithium, PTH and calcium levels do return to within the normal range, as was evident in our patient.
